# Improving educational environment in medical colleges through transactional analysis practice of teachers

**DOI:** 10.12688/f1000research.1-24.v1

**Published:** 2012-10-09

**Authors:** Marina Rajan, Thomas Chacko

**Affiliations:** 1 Malankara Orthodox Syrian Church Medical College, Kolenchery, Kerala Pin, 682311, India; 2 Faimer Regional Institute, PSG Medical College, Coimbatore, 641004, India

## Abstract

**Context:** A FAIMER (Foundation for Advancement in International Medical Education and Research) fellow organized a comprehensive faculty development program to improve faculty awareness resulting in changed teaching practices and better teacher student relationships using Transactional Analysis (TA). Practicing TA tools help development of ‘awareness’ about intrapersonal and interpersonal processes.

**Objectives:**

- To improve self-awareness among medical educators.
- To bring about self-directed change in practices among medical educators.
- To assess usefulness of TA tools for the same.

**Methods:** An experienced trainer conducted a basic course (12 hours) in TA for faculty members. The PAC model of personality structure, functional fluency model of personal functioning, stroke theory on motivation, passivity and script theories of adult functional styles were taught experientially with examples from the Medical Education Scenario. Self-reported improvement in awareness and changes in practices were assessed immediately after, at three months, and one year after training.

**Findings:** The mean improvement in self-'awareness' is 13.3% (95% C.I 9.3-17.2) among nineteen participants. This persists one year after training. Changes in practices within a year include, collecting feedback, new teaching styles and better relationship with students.

**Discussion and Conclusions:** These findings demonstrate sustainable and measurable improvement in self-awareness by practice of TA tools. Improvement in self-'awareness' of faculty resulted in self-directed changes in teaching practices. Medical faculty has judged the TA tools effective for improving self-awareness leading to self-directed changes.

## Context

There is an increasing awareness about the importance of educational environment in bringing about effective learning. "The learning environment created in the training group is crucial in helping trainees take risks while feeling supported" ^1^. Student’s perception of learning, their teachers, the learning atmosphere, and their academic and social self-perceptions are major components of the DREEM (Dundee Ready Education Environment) questionnaire developed for studying the environment of medical education ^2^. Many medical colleges in India are now taking initiatives to find out remedial measures to be taken to improve the learning environment ^3^.

Developing trusting relationships is one of the components of a model for self-directed learning ^1^. The need for a comprehensive faculty development initiative in institutions is emphasized among the various strategies that are directed to improve teaching practices in medical schools. Faculty development projects are hence included for Curriculum Innovation Projects in FAIMER Fellowship programs. A "Comprehensive faculty development, which is more important today than ever before, empowers faculty members to excel as educators and to create vibrant academic communities that value teaching and learning" ^4^.

**Figure 1.**
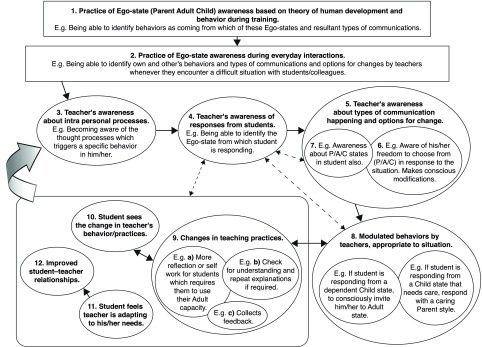
Dynamics of self-awareness and self-directed behavior change.

Comprehensive faculty development could be done by using the Transactional Analysis (TA) theory of personality development (see What is TA). It can be the basis for bringing about faculty development by facilitating their understanding of their own teaching styles by practice of the TA tools. Transactional Analysis belongs to the humanistic school of psychology ^1^. The International Transactional Analysis Association explains "Educational transactional analysis is used by practitioners working in training centers, preschools, elementary and high schools, universities, and institutions that prepare teachers and trainers as well as in support of learners of all ages to thrive within their families, organizations, and communities." (see About TA Transactional Analysis), TA describes a theory of the structure of personality – Ego state (P-A-C-) model (see Key Ideas in TA) and how people communicate with each other based on this structure. Since education is an area of intensive interpersonal processes, practicing self-awareness using this model is known to help develop self-awareness ( *herein after referred to as ‘awareness’*) ^5^. The dynamics of how self-awareness leads to self-directed improvement in teaching is illustrated in figure 1.

A survey conducted among students in our institution to find out their perceptions about the existing system of medical education and suggestions for improvement revealed many lacunae in the teacher-student relationships that form a barrier to learning and thus contribute to a poor learning environment (Marina Rajan Joseph, Student Perceptions about Learning Environment in a New Medical College in Kerala, Unpublished document). In this context, it was felt that an educational intervention study among the faculty to initiate interest among them about the need for an improvement in the learning environment within the institution is necessary. The author chose this as a Curriculum Innovation Project for her FAIMER fellowship.

## Objectives

1. To increase personal ‘awareness’ about the interpersonal processes that takes place during teaching-learning among medical educators.

2. To bring about a self-directed changes in the behavior of the teachers that makes them more student-centered and thereby contributing to a healthy educational environment.

3. To assess the usefulness of the TA tools for the above purposes.

It was assumed that an improved self-awareness about the teaching learning processes and teacher student interactions among teachers would bring about a better understanding of lacunae in teacher student relationships and motivate teachers for self-directed corrective measures thereby resulting in an improved learning environment for the students.

## Methods

### Ethical clearance

The project was presented to the institutional ethics committee and ratification obtained. Verbal informed consent was obtained from the participants.

### Study design

An interventional study without a control group has been used. The author, a trained Transactional Analysis (Edu) trainer with five years of experience in training, conducted a course in TA with focus on intrapersonal and interpersonal processes, for medical faculty members in our college.

### Sample size

Since it was an explorative study all faculty members who volunteered were included in the study.

### Program content

The content of the program was designed based on the framework of 101 (Basic) courses in Transactional Analysis ^7^. This included the Okay-Okay philosophy of TA, Structure of personality (the ego states or Parent – Adult – Child model), the Functional Fluency model, a new model in TA developed from the ego state model specifically for educators and trainers ^8^, stroke theory on motivation- a theory which states that ‘Stroking’ or ‘stimulation’ is essential for health and that wherever positive stroking is lacking, there persons may seek negative strokes to affirm their existence- ^5^, passivity theory which teaches about passive behavior which are said to be the barriers to effective problem solving and script theory which teaches about how early childhood decisions affect our style of function as adults.

### Session delivery

Two colleagues trained in TA helped the author with the sessions, data collection and also provided structured and concurrent feedback to the trainer. Their feedbacks on the trainer and participants helped in maintaining objectivity during the training program. The program extended over 12 hours (2 days). The course was conducted ‘contractually’ ( *negotiating between the expectations of participants and what the trainer got to offer*) and using ‘open honest transactions’ ( *objectively stating what is expected, and appropriate, descriptive rather than prescriptive feed backs*) the two principles of TA practice.

The program was experiential with exercises and practice sessions for developing personal ‘awareness’ through practice of the Ego state tool ^9^, stroking practice and role plays on types of interpersonal transactions. There were many one to one, small groups and large group discussions with opportunity for reflections. All theoretical aspects were illustrated using examples and case studies from everyday processes in Medical education. There participants introspected, shared insights and planned for future action based on insights developed.

### Students’ perceptions

Student’s perceptions about the present system of teaching and suggestions for improvement (students wanted teachers to be friendlier and teach more interactively and from practical experience) were also communicated to the teachers.

### Evaluation

Self-assessment of the participant’s ‘awareness’ before training, immediately after, at three months and one year after the training program were collected using an adapted version of a retro pre-behavior assessment tool available free on the Business Balls website. This included awareness’ in eighteen different areas of interpersonal relations.

### Analysis

The improvement in awareness was assessed quantitatively using Epi Info. Mean improvement in awareness of the group at three different times after training and mean improvement in awareness about specific areas of behavior were calculated and compared. Program feedback and evaluation on the usefulness of the tool was also collected using a post test questionnaire. Consensus on the usefulness of the TA theory and its tools for improving self-awareness was calculated using the Consensus index ^10^. Self-reported changes in practices have also been quantified.

Qualitative analyses of the group discussions and recommendations from action plans have also been included in the discussion.

## Results

Nineteen faculty members of different age and experience participated in the training (Table 1). After 3 months only 12 members and after 1 year only 11 members were available for follow up. Table 2 to Table 5 show the findings in mean improvement in awareness, areas in interpersonal processes where improvement has occurred, consensus on the usefulness of the TA tools and self-directed changes in practices resulting from awareness.

**Table 1.** Showing the demographic profile of the participants.

**Educational** **qualifications**		**Gender** **distribution**		**Departments/Subjects** **taught**		**Age pattern**		**Teaching experience**	
MBBS – MD/MS – Dip NB	6 12 1	Male – Female –	10 9	Physiology – Forensic – Com. Medicine – Internal Medicine – Surgery – Orthopedics – Obs. Gyne – Pediatrics – Dermatology –	2 3 3 2 1 3 2 2 1	<30 Years 31–40 years 41–50 years – 51–60 year – >60 years –	3 7 3 4 2	<1 year – 2–5 years 6–10 year >15 years	4 6 7 2
Total –	19		19		19		19		19

**Table 2.** Showing the self-perception of improvement in mean awareness by participants at different times after training.

**Time of assessment**	**% score** **before exposure** **to TA training**	**% score** **after exposure** **to TA training**	**Difference in** **score between** **before and after**	**95%** confidence limits*	**P value**
**Immediately after** **training n-(19)**	54.91	68.27	13.36	9.3 – 17.2	> 0.001 for 18 d.f.
**3 months** **later n-12**	53.28	65.55	12.26	8.94 – 15.58	> 0.001 for 11 d.f.
**1 year** **later n-11**	46.31	60.4	14.09	9.87 – 18.31	> 0.001 for 10 d.f.

* As can be seen from the 95% confidence limits of each value there is no statistically significant difference between the mean improvements recorded immediately after training, three months later or one year later.

**Table 3.** Showing the areas of Personal Awareness showing greatest improvement in at different stages of assessment. (Q. no. of the assessment sheet).

**Areas of awareness**	**% improvement** **in score immediately** **after training**	**% improvement** **persisting three** **months after training**	**% improvement** **persisting 1 year** **after training**
(Q.no.1) Being aware of my own behavior with others	16.85%	15.8%	22.72%
Q.no.3 Being aware of reactions of others to my behavior	15.26%	19.1%	21.8%
Q.no.4 ‘Being aware of my reaction to the behavior of others’	21.6%,	15%	13.6%
Q.no.14 ‘Being aware of what behavior modification I need to do’	19.5%,	16.7%	16.36%
Q.no.15 ‘Knowing how to modify my behavior’	19.5%.	14.1%	14.54%
Q. no.18 The general level of my interpersonal skills with others	16.3%	12.5%	20%

**Table 4.** Showing consensus of participants on the usefulness of different TA tools.

**Types of tools**	**Consensus %**
PAC Model for practicing self-awareness	88.7%
Functional fluency model for flexibility in functioning	84.8%
Usefulness of TA tools for better Teacher student relationships	81.2%
Usefulness of the TA tools for medical teacher’s training	62.4%* *73.73% one year after training

**Table 5.** Showing the changes in practices of faculty one year after training.

**Changes in practices**	**No of persons** **n-11**	**%**
Conscious change in responses while communicating	11	100
Changes in teaching styles	9	81.8
Collecting feedback from students	6	54.5
Improved relationship with students	8	72.7
Collecting feedback from peers	0	0

## Discussion

The mean improvement in self-awareness immediately after training persists at three months and one year after training (Table 2). It reflects the lasting quality of the awareness generated which is behavioral, a Kirkpatrick level three benefit (see Kirkpatrick Philosophy). Though other researchers ^5^ have reported improvement in self-awareness after learning TA, there are not many reports on measuring and grading this awareness. In this study the greatest improvement in awareness was reported in three areas namely ‘being aware of my reaction to the behavior of others’, ‘being aware of what behavior modification I need to do’, and ‘knowing how to modify my behavior’ (Table 3). This is a very positive outcome of the training because in improving teacher student relationships, it is these three awareness competencies that help the teacher modulate behavior in the here and now of the teacher student interaction. It is also interesting that as time elapse after training, practice of awareness over a year has consistently improved the faculty’s self-perception about their ‘general interpersonal skills’. Research among student teachers has demonstrated that becoming aware of their own attitudes, beliefs and cognition helped them to achieve specific aims in professional practice ^5^. It has been demonstrated among Russian university students that use of Transactional Analysis by them helped them to gain a high level of knowledge about effective relationships and ability to use this knowledge in their professional and personal lives ^11^. The group’s consensus on usefulness of TA for medical teacher’s training has increased one year after training (Table 4). It probably reflects their personal experience of practicing the tools and perceived benefits thereof. The high degree of consensus among participants who are all doctors, on the usefulness of TA tools for Medical Teachers training, and on the usefulness of the PAC and ‘Functional Fluency’ models prompts future studies on these tools.

During discussions the participants connected needs expressed by the students to the stroke theory on motivation. TA research work with College students has shown that greatest growth was reported in the area of stroking ^12^. They identified lack of positive stroking in the medical education scenario as a probable reason for the lack of motivation they observed among many students, particularly considering the fact that in India students come to the medical college straight from schools. Positive stroking and Open Honest Transactions were identified by the participants as the most practical TA tools for improving teacher student relationships.

Education and psychology has a shared history and "It has been the humanistic psychologists who have grappled mostly with the problems of learning" ^1^. She quotes Malcom Knowles who formulated the theory of adult learning and ‘andragogy’, as saying that "Some of the most important contributions to learning theory have come from psychotherapy". Carl Rogers famous for his innovative ‘Client-centered therapy’ applied the same to education and said that education is "facilitation of learning" and educator is "facilitator of learning" thus bringing in a ‘student centered’ approach to education. ^13^. The transition from use of the term ‘Pedagogy’ to ‘Andragogy’ also reflects a transition from teacher-centered education to student-centered education ^14^. The four desirable factors in teachers namely, ‘warmth, enthusiasm, use of discovery-learning methods, and high level of cognitive organization’ ^15^ are also qualities of educators who are student centered. Many faculty members in this study have adopted changes in similar practices within one year of attending the Transactional Analysis training (Table 5). It shows that comprehensive faculty development such as using the TA approach fosters development of many of the qualities of student centered among medical educators.

The participants developed an action plan at the end of the training. They requested more organized and formal training in pedagogical methods along with more inputs on interpersonal skills. They volunteered to organize an effectively functioning Medical Education unit that not only sharpens pedagogical skills but also leads to a better understanding of students, their learning processes and create a favorable educational environment in the institution.

## Conclusion

These results suggest that practice of the ego state awareness using the PAC model helps to improve self-awareness and sustains it even up to a year after training. This awareness in turn is helping teachers to become aware of their own and student’s behavior in different situations and makes appropriate modifications. This in turn helps them to practice new teaching styles and improve teacher student relationships. Participants reported increasing confidence in the TA tools for medical teacher’s training. It suggests that continued and consistent inputs using the same may remarkably improve personal ‘awareness’ among teachers and thus the teacher-student relationship, leading to an improvement in the educational environment vital for promoting student learning.
