# Supplementary material for: Improving educational environment in medical colleges through transactional analysis practice of teachers
Source: F1000Res. 2012 Oct 9;1:24. [Version 1] doi: 10.12688/f1000research.1-24.v1 (PMC3751144; doi:10.12688/f1000research.1-24.v1)
Supplement: Peer review history

# Peer review: Referee response for version 1

**Published:** 2012-10-26

**Authors:** Kirsty Forrest

**Affiliations:** Research and Evaluation Study Skills (RESS formally SSC) Co-Chair, University of Leeds, Leeds, UK

This article describes a practical intervention for faculty development. Faculty development is currently the poor relation within medical education, and this study provides evidence of how to improve student learning by improving educator insight.

I have read this submission. I believe that I have an appropriate level of expertise to confirm that it is of an acceptable scientific standard.

# Peer review: Referee response for version 1

**Published:** 2012-10-25

**Authors:** Judy McKimm

**Affiliations:** College of Medicine, University of Swansea, Swansea, UK

This paper provides an interesting write up of a research project looking at how to introduce Transactional Analysis (TA) into faculty development activities to improve the learning environment.

The methods are sound and relevant to the topic being researched and the findings consistent with other similar studies, giving evidence-based recommendations about using TA to improve practice.

I have read this submission. I believe that I have an appropriate level of expertise to confirm that it is of an acceptable scientific standard.
